# Targeting S100B with Peptides Encoding Intrinsic Aggregation-Prone Sequence Segments

**DOI:** 10.3390/molecules26020440

**Published:** 2021-01-15

**Authors:** Joana S. Cristóvão, Mariana A. Romão, Rodrigo Gallardo, Joost Schymkowitz, Frederic Rousseau, Cláudio M. Gomes

**Affiliations:** 1 Biosystems and Integrative Sciences Institute, Faculdade de Ciências, Universidade Lisboa, 1749-016 Lisbon, Portugal; jmcristovao@fc.ul.pt (J.S.C.); maromao@fc.ul.pt (M.A.R.); 2 Departamento de Química e Bioquímica, Faculdade de Ciências, Universidade Lisboa, 1749-016 Lisbon, Portugal; 3 VIB Switch Laboratory, Flanders Institute for Biotechnology (VIB), 3000 Leuven, Belgium; rodrigo.gallardo@switch.vib-kuleuven.be; 4 Switch Laboratory, Department of Cellular and Molecular Medicine, KU Leuven, Herestraat 49, PB 802, 3000 Leuven, Belgium

**Keywords:** S100 proteins, protein aggregation, amyloid, protein biophysics, structural biology

## Abstract

S100 proteins assume a diversity of oligomeric states including large order self-assemblies, with an impact on protein structure and function. Previous work has uncovered that S100 proteins, including S100B, are prone to undergo β-aggregation under destabilizing conditions. This propensity is encoded in aggregation-prone regions (APR) mainly located in segments at the homodimer interface, and which are therefore mostly shielded from the solvent and from deleterious interactions, under native conditions. As in other systems, this characteristic may be used to develop peptides with pharmacological potential that selectively induce the aggregation of S100B through homotypic interactions with its APRs, resulting in functional inhibition through a loss of function. Here we report initial studies towards this goal. We applied the TANGO algorithm to identify specific APR segments in S100B helix IV and used this information to design and synthesize S100B-derived APR peptides. We then combined fluorescence spectroscopy, transmission electron microscopy, biolayer interferometry, and aggregation kinetics and determined that the synthetic peptides have strong aggregation propensity, interact with S100B, and may promote co-aggregation reactions. In this framework, we discuss the considerable potential of such APR-derived peptides to act pharmacologically over S100B in numerous physiological and pathological conditions, for instance as modifiers of the S100B interactome or as promoters of S100B inactivation by selective aggregation.

## 1. Introduction

S100 proteins are a family of EF-hand Ca^2+^-binding signaling proteins that have evolved in vertebrates to carry out a multitude of functions related to proliferation, differentiation, cell apoptosis, migration, energy metabolism, calcium homeostasis, and inflammation, under physiological and pathological conditions [1,2]. These small (~12 kDa) dimeric proteins are extremely versatile, and their biological activity is regulated by expression levels, metalation state, cellular location (intra vs extracellular), and quaternary structure and assembly state [3,4]. Notably, S100B is one of the S100 proteins that has been implicated in cancer and in neurodegenerative diseases, and therefore there is ample interest in the pharmacological modulation of its activity [5,6].

Compounds such as pentamidine have promising therapeutic activities as inhibitors of S100B function in multiple pathological scenarios [7,8]. For instance, pentamidine inhibits the interaction between p53 and S100B, thus reactivating the transcriptional activity of p53 apoptosis in cancer cells; also, pentamidine decreases gliosis and neuroinflammation in a mouse model of Aβ-induced Alzheimer’s Disease (AD) suggesting an inhibitory activity over the interaction between S100B and RAGE (receptor for advanced glycation end-products) [9], similarly to other S100 proteins [10]. Also, S100B protein functions are frequently concentration-dependent, being protective at low concentrations and deleterious at high levels. The involvement of S100B in AD is one such example: in late disease stages when it is overexpressed and secreted by astrocytes, S100B acts as a pro-inflammatory cytokine that promotes gliosis and aggravates neuroinflammation as a response to the accumulation of amyloid beta deposits [11]; however, at earlier inflammatory stages, when the relative levels of Aβ42 and S100B are still increasing, S100B exerts a protective chaperone-like activity engaging in interactions with Aβ42 that inhibit its aggregation and mitigate amyloid toxicity [12,13]. It is therefore also useful to have drugs that are not only able to completely inhibit S100B activities but that are also able to regulate its levels in cells.

In this respect, approaches based on the use of aggregation-prone peptides as tools to selectively inactive or modulate the levels of a given target protein, hold the potential to develop such sophisticated therapeutic biologics. The strategy of selective aggregation of globular proteins mediated by peptides is based on aggregation seeding, which is a process driven by so-called aggregation-prone regions (APRs). These are short amino acid stretches that are present across proteomes [14] and which dictate the propensity for β-aggregation being extensively found in globular proteins [15]. These APRs engage in homotypic interactions resulting in the formation of tightly packed intermolecular amyloid structures. Synthetic biology approaches have demonstrated that peptides derived from APRs present in a given protein are able to interact selectively with that specific protein, causing its inactivation through aggregation. This approach has been established in several systems, and examples include the use of APRs to generate selective knockdowns in plants [16] or as anti-bacterial agents with antibiotic properties in bacteria, but not in infected mammalian cells [17].

S100 proteins are amenable targets to develop APR-based functional modulators with pharmacological potential. Previous reports showed that S100 proteins have aggregation-prone regions at the dimer interface that are flanked by intrinsically disordered regions [18,19]. Upon destabilization, these regions become solvent-exposed, homotypic interactions take place and β-aggregation evolves [20,21]. Here, we explore this property to design, synthesize, and characterize APR peptides inspired in S100B, as a first approach towards the future development of pharmacologically active biologics capable of targeting and modulating S100B levels and functions. For this, we developed a mini library of three peptides inspired in APRs within helix IV of S100B, which we characterized with respect to their aggregation propensities, interaction with S100B, and in vitro co-aggregation.

## 2. Results and Discussion

### 2.1. S100B-Derived APR Peptides: Design and Synthesis

**Figure 1:**
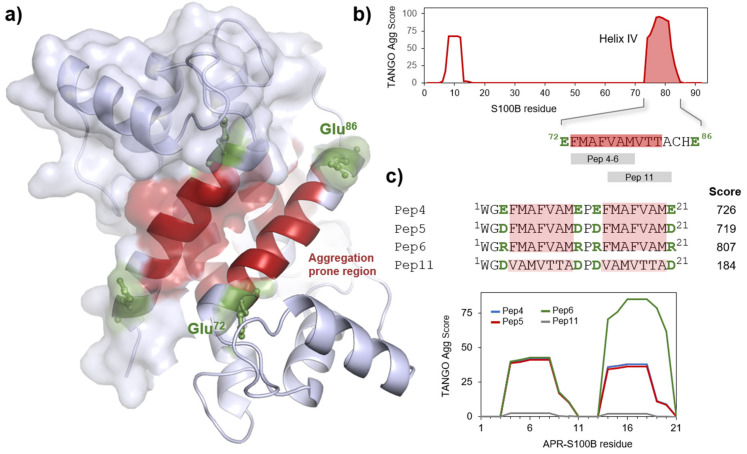
S100B structure and designed aggregation-prone peptides. (**a**) Structure of the human S100B dimer with the aggregation-prone helix IV marked in red and its capping Glu gatekeeper residues depicted in green; one of the subunits is marked with surface contour. Figure made with Pymol [25] based on the Protein Data Bank (PDB) entry 3d0y; (**b**) Plot of TANGO scores of human S100B, highlighting helix IV and its APR sequence; (**c**) Sequence of the designed APR-S100B peptides with the tandem APR highlighted in red, gatekeepers in bold green and plots of TANGO scores.

We employed TANGO, a statistical algorithm for predicting aggregation-prone sequences [22], to analyze the sequence of human S100B and determined that the region with the highest aggregation propensity locates within helix IV, in residues 73 to 85 (Figure 1). Flaking this segment are two glutamate residues (Glu-72 and Glu-86), which act as gatekeepers. Aggregation gatekeepers are residues observed at the sides of these aggregation-prone sequence segments that protect from aggregation [15,23,24]; such residues are frequently charged and counteract aggregation through repulsive effects. We used the information from TANGO to identify sequences that comprise at least six successive amino acids with high aggregation scores, which guided the design of four representative peptides within this region (Figure 1c). Each APR-S100B peptide is composed of a PEG2-biotin and two identical repeats of the β-aggregating sequence flanked by different gatekeeper residues that act as solubilizing moieties. The aim of including tandem sequences was to amplify the aggregation potential by repeating APR patterns. In the designed APR-S100B peptides, which were inspired in sequences at the beginning of helix IV (Pep4 to 6), we made variants that include not only the natural gatekeeper (Glu) but also other charged residues (Asp and Arg). Including gatekeepers in these constructs is critical for harnessing the aggregation potential through charge repulsions, as otherwise, the APR-S100B peptides would promptly fibrillate, rendering them of little use to establish regulatory interactions with the S100B target. One additional peptide corresponding to the last stretch of helix IV was also synthesized to further scan this region (Pep11). This peptide has the lowest TANGO aggregation score and thus serves also as a control. It is important to note that the reason for which we did not synthesize peptides with no gatekeeper residues is that such peptides would not be suitable controls as they would form stable amyloid fibrils even faster [15], and therefore would not be available to engage in homotropic interactions with helix IV in S100B, as we seek to test. All peptides were obtained by solid phase peptide synthesis at good yields and purity, as analyzed by High-performance liquid chromatography (HPLC) (Figure S1).

### 2.2. Aggregation Propensity of S100B-Derived APR Peptides

**Figure 2:**
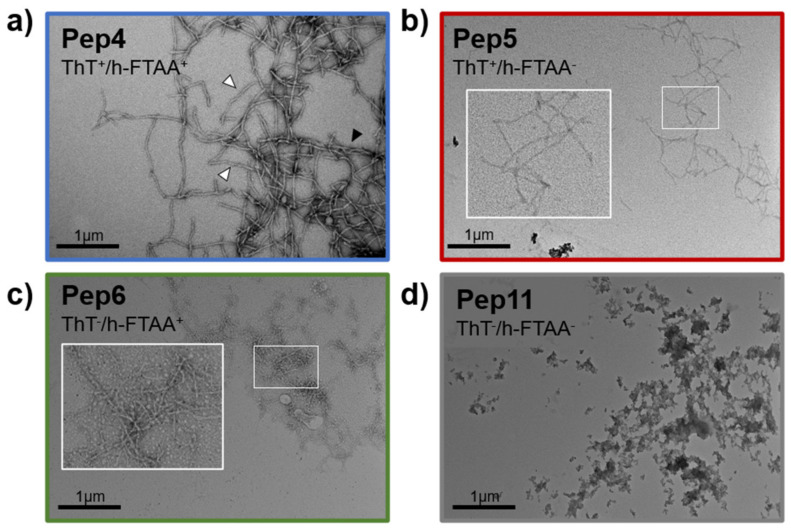
Transmission electron microscopy of aggregates formed by APR-S100B peptides. (**a**) APR-Scheme 100. B-Pep4, black arrowhead denotes fibril side stacking and white arrowheads illustrate twisted fibrils; (**b**) APR-S100B-Pep5; (**c**) APR-S100B-Pep6 and (**d**) APR-S100B-Pep11. White boxes indicate magnifications illustrating details of fibril networks. Superscripts + and − in ThT and h-FTAA indicate, respectively, positivity or negativity of aggregates in respect to these amyloid-detecting dyes. Aggregates were generated following incubation of peptides (200 µM) for 24 h at 37 °C.

We then proceeded with the characterization of the aggregation propensity of the synthesized peptides to test if the computed aggregation propensity translates into the formation of fibrillar aggregates. After solubilization and filtration, all the peptides were polydispersed in a solution while kept in ice. However, they become more polydisperse over time suggesting that the formation of aggregates took place. We resorted to thioflavin-T (ThT) fluorescence emission to monitor if the formed aggregates have an amyloidogenic character, as ThT is a well-established fluorescent reporter for cross β-rich aggregates [26]. For this, we incubated APR-S100B peptides (200 µM) in 50 mM (4-(2-hydroxyethyl)-1-piperazineethanesulfonic acid) (HEPES) pH 7.4 at room temperature for up to 24 h and determined that only two peptides are clearly ThT positive (Pep4 and Pep5). Given the high aggregation propensity encoded within most of these sequences, we hypothesized that lack of ThT binding might indicate that the peptides would be forming polymorphic fibrils that might be undetected by ThT, whose limitations are well known. We thus tested heptamer-formyl thiophene acetic acid (h-FTAA), which is a dye from the family of luminescent conjugated oligothiophenes (LCOs) [27], that is known to recognize multiple types of β-aggregates. Indeed, Pep6 tested positive for this fluorophore suggesting that it forms a different type of amyloid morphotype. On the other hand, Pep11 was negative for both ThT and h-FTAA, in agreement with its low aggregation propensity score. To investigate fibril structure further, we employed transmission electron microscopy (TEM) to analyze the morphology of aggregates formed by the APR-S100B peptides at identical concentrations (200 µM) and incubation times (24 h at 37 °C) (Figure 2). We noted that, indeed, the analyzed peptides form aggregates with different morphologies as a result of polymorphic variability. APR-S100B-Pep4 forms thick amyloid-like fibers denoting some twisting which seems to result from side-by-side adhesion of fibrils (Figure 2a). APR-S100B-Pep5 and Pep6 form smaller and thinner fibers (Figure 2b,c) while Pep11 generates amorphous aggregates. This confirms that the APR-S100B peptides derived from S100B helix IV core (Pep4 to Pep6) are highly amyloidogenic, while the control APR-S100B-Pep11 peptide, which has a low aggregation score, does not form organized self-assemblies. Amyloid fibrils are known to be highly polymorphic and heterogeneous, and studies in bulk such as those by TEM in which multiple morphological subtypes of fibrils that are present necessarily translate this aspect. The observed differences in the fibril morphology and binding of amyloid-sensitive dyes illustrate the relevance of gatekeeper residues in determining peptide self-assembly as well as the morphology of the formed fibrillar materials, establishing that S100B-APR Pep 4, 5, and 6 are fibril forming, in agreement with TANGO predictions.

### 2.3. APR-Peptides Bind to S100B, which Modulates Their Aggregation Propensity

**Figure 3:**
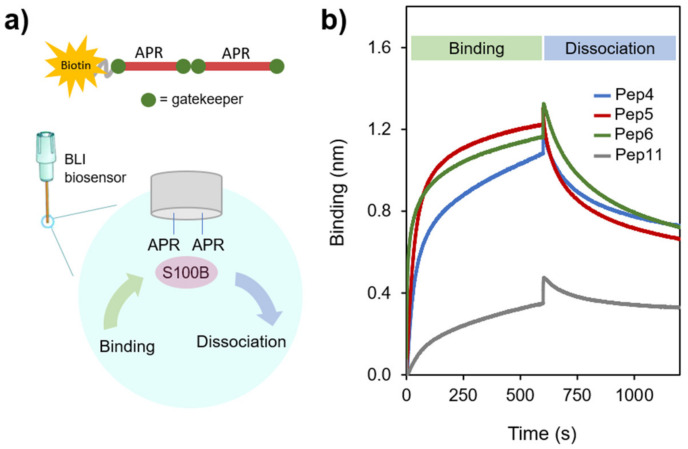
Biolayer interferometry analysis of the interaction between APR peptides and S100B. (**a**) The synthetic APR-S100B peptides were C-terminal acetylated and linked to a PEG-biotin tag which served to link the peptides to a streptavidin BLI biosensor; (**b**) BLI binding interferograms representative of at least 3 independent traces. Experiments were carried out at a fixed S100B concentration (50 μM) with 50 mM HEPES pH 7.4, 150 mM NaCl, 0.01% Tween 20 at 25 °C.

To establish the potential use of APR-S100B peptides as modulators of S100B function, we first investigated if the peptides bind S100B and then if S100B influences their aggregation. To further characterize the interaction between the APR peptides and S100B, we employed biolayer interferometry (BLI), which is a label-free optical technique for measuring macromolecular interactions through the analysis of interference patterns of white light reflected from the surface of a biosensor tip to which one of the ligands is attached [28]. This technique allows thus to infer interactions by assessing the kinetics of binding and dissociation by measuring the shift (in nm) in the interference pattern during the interaction. We used high precision Streptavidin biosensors to record the interaction of S100B to the biotinylated peptide (Figure 3a). In a typical experiment, the biotinylated peptide is bound to the biosensor, which is washed to remove the excess of unbound peptide and then dipped into a protein solution to determine the binding kinetics, followed by a dissociation step versus buffer. Since some aggregation of APR-S100B peptides during the time course of the BLI experiments could not be excluded, estimation of binding affinities was not pursued, as this would require accurate concentration-dependence studies. The goal of the BLI experiment was thus to demonstrate that the APR-S100B peptides with high TANGO scores do interact with S100B, in a single condition, in excess of peptides and S100B. For this, we optimized an assay using tetrameric S100B as it generated larger amplitudes of binding than the dimer (Figure 3b). Both forms are physiologically relevant and have similar exposure of the corresponding segments in helix IV [29], which allow templated binding with the synthetic peptides under study. Using this approach, we determined that Pep4, Pep5, and Pep6 bind to S100B with comparable amplitudes denoting a strong interaction, while decreased binding of Pep11 to S100B is observed, as expected for a control peptide with a low TANGO score.

**Figure 4:**
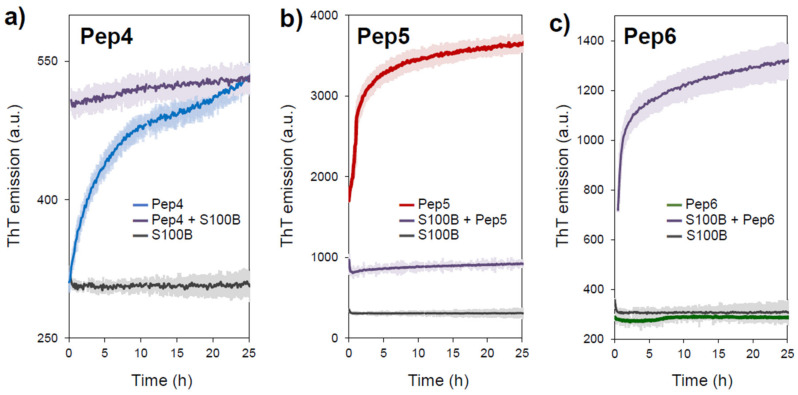
Aggregation kinetics of APR-S100B peptides in the presence of S100B. The time course of ThT emission was recorded as a function of time for (**a**) APR-S100B-Pep4, (**b**) APR-S100B-Pep5, (**c**) APR-S100B-Pep6 at 35 μM for each peptide in 50 mM HEPES pH 7.4 at 37 °C with agitation at 86 rpm, in the absence and in the presence of equimolar S100B. Traces depict averaged curves of 3 independent experiments with standard deviation marked as colored shades.

We then tested if the interaction between the APR-peptides and S100B might induce its aggregation. This is an important feature to establish if these peptides can be employed as non-genetic knockouts of S100B by causing its selective inactivation through targeted aggregation, as established for other systems [16,17]. To test this possibility, we carried out ThT-monitored aggregation kinetics experiments of 35 μM APR-S100B peptides Pep4, Pep5, and Pep6 in the absence and in the presence of equimolar S100B, during 25 h (Figure 4). S100B does not bind ThT and its fluorescence intensity does not change over time, as previously reported [12].

The peptide APR-S100B-Pep4 promptly forms ThT positive aggregates, with no lag phase, as expected for a highly amyloidogenic fragment, and ThT emission reaches a plateau approximately above 10 h. Interestingly, the presence of S100B results in an instantaneous increase in ThT fluorescence, at levels comparable to those of the plateau stage reached in its absence, suggesting that the S100B:Pep4 interaction enhances the formation of aggregates (Figure 4a). The same sharp increase in ThT emission is observed in the aggregation kinetics of APR-S100B-Pep5 but, in this case, the presence of S100B abolishes aggregation (Figure 4b). This observation is compatible with an inhibitory interaction between Pep5 and S100B that mitigates the formation of ThT positive fibrillar materials. These observations are compatible with previous ThT fluorescence emission and TEM data (Figure 2). A very interesting effect is observed with peptide APR-S100B-Pep6: we observed that this peptide is ThT negative although it forms fibrillar materials (Figure 2c) and this is consistently observed in the aggregation kinetics (Figure 4c). However, the combination of APR-S100B-Pep6 with S100B results in the formation of ThT positive aggregates (Figure 4c). This observation is compatible with co-aggregation phenomena described for other models, in which APR peptides can selectively aggregate the proteins from which they were derived.

## 3. Materials and Methods

### 3.1. S100B Expression and Purification

All reagents were of the highest grade commercially available. A Chelex resin (Bio-Rad, Amadora, Portugal) was used to remove contaminant trace metals from all solutions. Human S100B was expressed in *E. coli* (BL21 DE3) cells and purified to homogeneity using previously established protocol [30]. ApoS100B was prepared with incubation at 37 °C for 2 h with a 300-fold excess of Dithiothreitol (DTT) and 0.5 mM Ethylenediamine tetraacetic acid (EDTA) and eluted in a Superdex S75 Tricorn (GE Healthcare, Oeiras, Portugal) with 50 mM Tris Chelex pH 7.4.

### 3.2. Peptide Design and Synthesis

The β-aggregation-prone sequences were identified using TANGO. The criteria for selecting the two regions were the following: (a) the peptide stretch should have at least six successive amino acids with a TANGO score >5 and (b) the total score of the selected window should exceed >50, highly indicating APRs. The C-termini of these peptides were acetylated and linked to a PEG-biotin tag. Peptides were synthesized on an Applied Biosystems 430A synthesizer (Life Technologies Corp., Carlsbad, CA, USA) according to standard procedures or on a Hamilton Microlab 2200 (Hamilton, Reno, NV, USA) using Fmoc/2″-(1H-benzotriazole-1-yl)-1,1,3,3-tetramethyluronium hexafluorophosphate (HBTU) chemistry [31]. The purity of the synthesized peptides was assessed by HPLC. Peptides were routinely solubilized in 100 μL of 100 mM Ammonium Hydroxide, 150 μL of 50 mM HEPES pH 7.2 and 100 μL of 50 mM Acetic acid, and filtered. All peptide manipulations were done at −4 °C. Peptide concentration was measured by absorbance spectroscopy using an estimated ε (280 nm) = 5500 M^−1^ cm^−1^.

### 3.3. Fluorescence Spectroscopy

Fluorescence emission intensity of the APR-S10 peptides was measured in a plate reader (BMG, Fluostar Optima, Ortenberg, Germany) using the following fluorophores and conditions: ThT (λ_exc_ = 440 nm and λ_em_ = 480 nm) and h-FTAA (λ_exc_ = 480 nm and λ_em_ = 520 nm). For the analysis of ThT aggregation kinetics, peptides at 35 μM were incubated at 37 °C for up to 25 h, in 50 mM HEPES pH 7.4 with agitation at 86 rpm for 20 s before each cycle. S100B was also added at 35 μM. Real-time ThT fluorescence at 480 nm was recorded using a BMG Fluostar Optima (Ortenberg, Germany) fluorescence plate reader upon excitation at 440 nm.

### 3.4. Transmission Electron Microscopy (TEM)

For each sample, 7 μL aliquots of peptides, typically at 5% APR-S100B, were adsorbed for 1 min to formvar film coated on 400-mesh copper grids (Agar Scientific Ltd., Essex, UK), washed with water five times, and stained by contact with 2% uranyl acetate. The grids were examined using a JEM-2100 transmission electron microscope (Jeol, Tokyo, Japan) at 80 keV.3.5.

### 3.5. Bio-Layer Interferometry (BLITZ)

Protein:peptide interaction studies were performed on a Blitz instrument (ForteBio, Portsmouth, UK). The assays were done in 50 mM HEPES pH 7.4, 150 mM NaCl, 0.01% Tween20 at 25 °C and 1000 rpm. High precision Streptavidin biosensors (ForteBio, Portsmouth, UK) were used to record the interaction between S100B and the biotinylated peptides. A typical experiment consisted of the following steps: initial baseline for 30 s with the buffer; loading of biotinylated peptide to the biosensor for 120 s, followed by 180 s in buffer to remove the excess of peptide unbound to the sensor. Next, the sensor was dipped in a solution of S100B tetramer (50 μM) for the association step for 600 s and followed by dissociation with buffer for 600 s.

## 4. Conclusions

Given its involvement in several pathologies, S100B represents a unique potential target for pharmacological intervention. Indeed, there are several reported small molecule inhibitors such as pentamidine and heptamidine that target S100B and can modulate its biological function. Here we present a preliminary study on the development of peptides containing tandem repeats of APRs derived from S100B that can selectively target the protein and modulate its function through selective co-aggregation. We showed that the designed APR peptides undergo amyloid-type aggregation, confirming that the APR segments from S100B undergo homotropic interactions that result in the formation of fibrillar materials. From BLI interaction experiments, we also concluded that those interactions may be recreated between the synthetic peptides and the corresponding regions in folded S100B, as binding was detected. Finally, we established that those interactions reciprocally influence (co) aggregation phenomena. From aggregation kinetic experiments, we observed that APR-S100B peptides such as Pep6 have the potential to induce aggregation of S100B, while, for instance, Pep5 seems to engage in stabilizing interactions that inhibit its self-assembly. With this work, we therefore establish the proof of principle for the potential use of S100B-derived APR peptides as modulators of S100B function and bioavailability, which may constitute future promising tools for pharmacological interventions targeting S100B.

## Data Availability

All data is presented in the manuscript.

## Supplementary Materials

Figure S1: HPLC chromatograms of the synthesized APR-S100B peptides.

## Abbreviations

AD	Alzheimer’s Disease
Aβ	Amyloid-β Peptide
APR	Aggregation Prone Region
ThT	Thioflavin T
RAGE	Receptor Advanced Glycation End Product
