# Supplementary material for: Targeting S100B with Peptides Encoding Intrinsic Aggregation-Prone Sequence Segments
Source: Molecules. 2021 Jan 15;26(2):440. doi: 10.3390/molecules26020440 (PMC7830867; doi:10.3390/molecules26020440)
Supplement: Supplementary file 1 [file molecules-26-00440-s001.pdf]

Supplementary materials

Article

Targeting S100B with Peptides Encoding Intrinsic Aggregation-Prone Sequence Segments

Joana S. Cristóvão [1,2], Mariana A. Romão [1,2], Rodrigo Gallardo [3,4,†], Joost Schymkowitz [3,4,*], Frederic Rousseau [3,4,*] and Cláudio M. Gomes [1,2,*]

1 Biosystems and Integrative Sciences Institute, Faculdade de Ciências, Universidade Lisboa, 1749-016 Lisbon, Portugal; jmcristovao@fc.ul.pt (J.S.C.); maromao@fc.ul.pt (M.A.R.)
2 Departamento de Química e Bioquímica, Faculdade de Ciências, Universidade Lisboa, 1749-016 Lisbon, Portugal
3 VIB Switch Laboratory, Flanders Institute for Biotechnology (VIB), 3000 Leuven, Belgium; rodrigo.gallardo@switch.vib-kuleuven.be
4 Switch Laboratory, Department of Cellular and Molecular Medicine, KU Leuven, Herestraat 49, PB 802, 3000 Leuven, Belgium
* Correspondence: cmgomes@fc.ul.pt (C.M.G.); frederic.rousseau@kuleuven.vib.be (F.R.); joost.schymkowitz@kuleuven.vib.be (J.S.)
† Current address: Astbury Centre for Structural Molecular Biology, School of Molecular and Cellular Biology, University of Leeds, Leeds LS2 9JT, UK

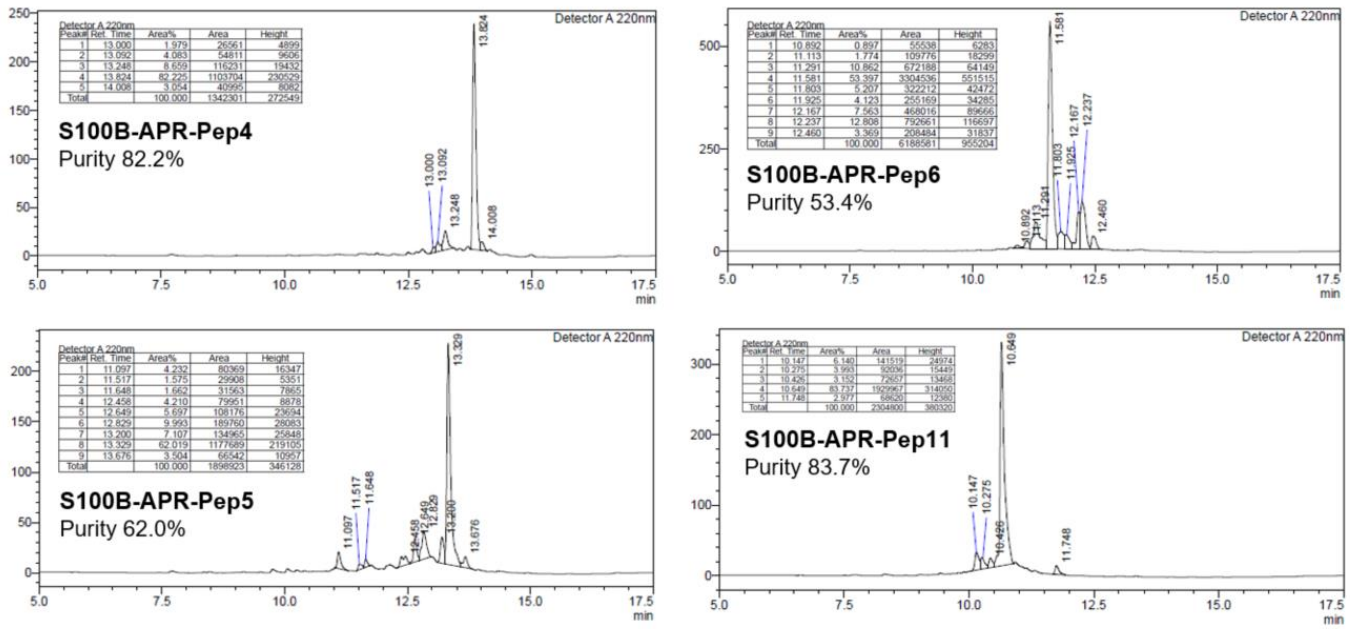

Figure S1. HPLC chromatograms of the synthesized APR-S100B peptides.
